# Women in surgery: a web-based survey on career strategies and career satisfaction

**DOI:** 10.1515/iss-2019-0016

**Published:** 2020-02-29

**Authors:** Sonia Radunz, Hülya Pustu, Katja Marx, Laura Mazilescu, Agnes Braun, Tamas Benkö, Mark Banysch, Gernot M. Kaiser

**Affiliations:** Department of General, Visceral and Transplant Surgery, University Hospital Essen, Essen, Germany; Department of General, Visceral and Transplant Surgery, University Hospital Münster, Alber-Schweitzer-Campus 1, 48140 Münster, Germany; Department of General and Visceral Surgery, St. Bernhard-Hospital Kamp-Lintfort, Kamp-Lintfort, Germany; MVZ Media Vita, Doctor’s Office for Surgery, Issum, Germany

**Keywords:** career, family, surgery, women, OR, operating room

## Abstract

Women represent the majority of medical students in several countries. In any surgical specialty and above all in surgical leadership positions, women still remain disproportionally underrepresented. The objective of this study was to investigate female surgeons’ career advancement and satisfaction with training. A standardized questionnaire was devised and sent out via the web-based survey tool SurveyMonkey^®^ to female surgeons in the German federal state of North Rhine-Westphalia. A total of 125 completed questionnaires were analyzed (response rate 40.8%). Female surgeons are at least largely (76%) satisfied with their surgical training. Increased time (>5 h/week) as the principal surgeon in the operating room significantly stimulates the satisfaction with the surgical training (86% vs. 68%, p = 0.0384). At the participants’ current workplace, the heads of departments are predominantly male surgeons (91%). Respondents not satisfied with their surgical training prefer a female head of department more frequently (24% vs. 2%, p = 0.0085). The majority of the respondents themselves aspire to become a consultant surgeon (56%), while only 12% intend to become a head of a department. Female surgeons aiming at leadership positions work overtime (≥50 h/week) significantly more frequently (81% vs. 57%, p = 0.0041). Favoritism of male colleagues is perceived by 34%. Respondents who do not perceive any preferential treatment are significantly more satisfied with their surgical training (88% vs. 57%, p = 0.0004). In conclusion, female surgeons seem positive about their career choice, once in the surgical profession, and aptly fill upcoming positions. Women interested in surgery are likely to pursue a surgical career despite the alleged workload, demonstrating the importance of professional self-fulfillment among female surgeons.

## Introduction

Surgical specialties have been suffering from a shortage of young recruits in several countries as fewer medical students pursue a surgical career [1]. In the past, the passion for the challenging profession of surgery seemed to have compensated for the drawbacks in (family) lifestyle [2]. Nowadays, lifestyle issues are the most cited factor influencing medical students’ career choices [3].

### Gender disparity in the surgical field

The majority of medical students nowadays are women. Among higher trainees, specialist doctors, and consultants in surgery, women are still underrepresented, comprising only 19% in Great Britain in January 2018 [4]. In further senior surgical career stages, the percentage of women is even lower, i.e. only 12% of consultant surgeons are women in Great Britain as of January 2018 [4]. The proportion of British women in surgical specialties increased from 24% in November 2009 to only 27% in November 2017 [4]. In Germany, there were 7983 active female surgeons in 2018, the equivalent of 21.1% of all active surgeons (2017: 7548 active female surgeons, 20.4% of all surgeons) [5], [6].

### Deterrents to a surgical career

It has been shown that women may be deterred from choosing a career in surgery because of numerous factors, e.g. work-life balance, small number of female role models, and gender discrimination [7]. Previous studies revealed a persistence of traditional gender roles: male graduates aimed at leading positions in hospital departments, while female medical graduates preferred outpatient clinics as their workplace [8], [9], [10], [11]. In addition, most medical graduates believe surgical specialties do not welcome female trainees [11]. Gender discrimination among surgeons has been discussed in the literature for many years as a justification for gender imbalances in the field of surgery [12]. Female academic surgeons are 10 times more likely to perceive gender-based discrimination [13], [14]. The most significant barriers for female academic surgeons are based on their experiences as women. These gender-based negative attitudes obstruct the career ambitions of female surgeons [14], [15]. A supporting network, i.e. a mentorship, is crucial so that female surgeons interested in an academic career have the opportunity for professional development. Mentoring significantly enhances the support in career advancement, especially in terms of networking [16].

### German surgical training

The German surgical training consists of 2 years of basic training and 4 years of higher surgical training. Upon a successful examination, one may then choose a position as a practice-based physician or one may continue working at a hospital as a fellow. To earn a proper title, supervised studies resulting in a doctoral thesis are required. The highest academic degree to be achieved is habilitation, i.e. postdoctoral university degree with lecture qualification. Habilitation may be compared to higher doctorates, such as the D.Sc. Habilitation remains the common German track to a professorship, which will only be granted after continued research work [17]. Depending on surgical expertise and research achievements, if applicable, a leadership position, e.g. consultant or head of department, may be granted.

### Study objective

It remains unknown whether women exhibit less interest or whether they find fewer chances for a surgical career. As the number of female medical graduates will further increase, this topic is crucial for the future of surgery. Consequently, we investigated the career strategies and satisfaction of German female surgeons.

## Materials and methods

We devised and tested a questionnaire exploring factors influencing the career intentions of female surgeons. We employed binomial, five-point Likert-scale questions as well as free-text questions. The local ethics committee (University Duisburg-Essen, file number 17-7780-BO) approved the study. We performed the study in accordance with the ethical standards as laid down in the 1964 Declaration of Helsinki and its later amendments.

Questionnaires were sent out electronically via the web-based survey tool SurveyMonkey^®^ to practicing female surgeons in departments of general and visceral surgery in the German federal state of North Rhine-Westphalia. All respondents gave informed consent to participate by completion of the survey. Participation was voluntary, anonymous, and without compensation.

The survey contained 33 questions on demographics, surgical education, career strategies, career satisfaction, and quality of life. The current workplace, the obtained surgical specialty training, and the satisfaction level with education were evaluated. Intended career advancement and appropriate support were assessed. Furthermore, we inquired the present and planned family lifestyle.

Data were collected using Microsoft Excel 2013 (Microsoft Corporation, Redmond, WA, USA), and statistical analysis was performed using GraphPad Prism version 6.07 for Windows (GraphPad Software, San Diego, CA, USA). Data are presented as absolute frequencies and percentages. Categorical variables were analyzed using Fisher’s exact test or the chi^2^ test, as appropriate. A p-value of ≤0.05 (two-tailed) was considered to be statistically significant.

## Results

### Respondents

We received 125 completed questionnaires, yielding a response rate of 40.8%. The respondents had a median age of 36 (26–61) years, covering a wide spectrum of surgical experience and at different stages of training.

### Surgical specialty training

**Figure 1:**
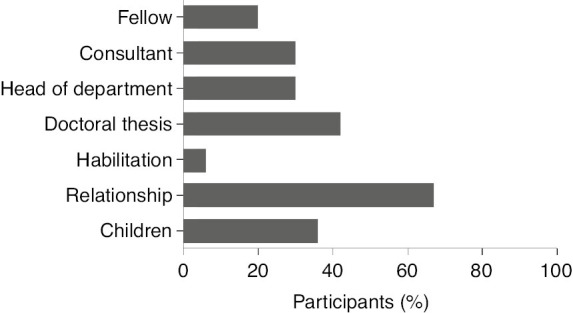
Professional and personal achievements of female surgeons.

At the time of the survey, 47.2% (n=59) of the participants had not yet completed their specialty training: 19.2% (n=24) were in first and second years of training, 11.2% (n=14) in third and fourth years, and 16.8% (n=21) in the fifth and sixth years of training. Respondents with completed specialty training were working as fellows (20.0%, n=25), consultants (29.6%, n=37), or heads of departments (3.2%, n=4) (see Figure 1).

Currently, 22.4% (n=28) of the respondents work at a university hospital, 25.6% (n=32) at a maximum care hospital, and 36% (n=45) at a community hospital. Sixteen percent (n=20) of the participants did not specify their current workplace. The head of department in the participants’ current workplace are male surgeons in 91.2% (n=114) cases and female surgeons in 8.8% (n=11) cases.

### Academic achievements

A doctoral thesis had been completed by 41.6% (n=52) of the participants and 6.4% (n=8) had achieved habilitation (Figure 1). Participants working at university hospitals had significantly more frequently completed a doctoral thesis and/or habilitation compared to female surgeons at maximum care or community hospitals (78.6%, 56.3%, and 44.4%, respectively; p=0.0164). Female surgeons who had completed a doctoral thesis and/or habilitation are significantly older [median 39 (26–61) vs. 34 (26–59) years, p=0.0174].

### Career strategies

**Figure 2:**
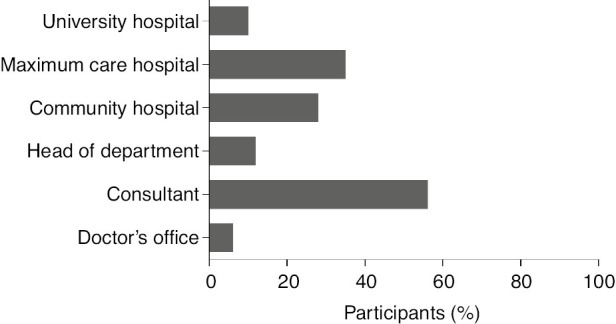
Career strategies of female surgeons: aspired work place and position.

The majority of the respondents aspire to become a consultant surgeon (56.0%, n=70); 12.0% (n=15) of the respondents intend being head of department, three of them aspiring to become chair of surgery. Only 5.6% (n=7) of the participants intend to set up their own doctor’s office (Figure 2). Sixteen percent (n=20) of the participants did not specify their further career plans. Among the present consultant surgeons, 67.6% (n=25) do not have any further career aspirations. Female surgeons aiming at leadership positions work overtime (≥50 h/week) statistically significantly more frequently than those without further career aspirations [81.0% (n=47) vs. 56.7% (n=38), p=0.0041].

In the future, only 10.4% (n=13) of the participants intend to work at a university hospital. The preferred workplace is a maximum care hospital among 35.2% (n=44) of the participants and 28.0% (n=35) intend to work at a community hospital (Figure 2). The remaining 26.4% (n=33) intend to work at an outpatient clinic/doctor’s office or did not specify their preferred working place. This represents a significant difference in current workplace and preferred workplace in the future (p=0.0080).

### Satisfaction with surgical training and salary

Of the participants, 34.3% (n=37) are fully satisfied with their surgical training and 41.7% (n=45) are largely satisfied, while 11.1% (n=12) are only partly satisfied and 12.9% (n=14) are dissatisfied with their training. Their income is fully satisfying for 36.0% (n=40), largely satisfying for 40.5% (n=45), partly satisfying for 10.8% (n=12), and not satisfying for 12.6% (n=14). Seventeen participants did not rate the level of satisfaction regarding their surgical training and their income.

There are no significant differences concerning satisfaction with their surgical training when accounting for age, status of specialty training, working position, working place, or academic achievements. Female surgeons who spend >5 h/week as the principal surgeon in the operating room (OR) are significantly more frequently satisfied with their surgical training (86.3% vs. 67.9%, p=0.0384). Time as an assistant surgeon in the OR does not influence satisfaction regarding surgical training. Female surgeons who are satisfied with their surgical training are more frequently satisfied with their income as well (79.3% vs. 56.9%, p=0.0354). Respondents not satisfied with their surgical training would prefer a female head of department significantly more frequently (23.81% vs. 2.44%, p=0.0085).

### Gender differences

The majority of the participants (52.8%, n=66) do not perceive any gender discrimination. Preferential treatment of male colleagues in general is perceived by 10.4% (n=13) of female surgeons. Preferential treatment of male colleagues by individual surgical teachers or regarding certain procedures is described by 23.2% (n=29). Respondents who do not perceive any preferential treatment are significantly more satisfied with their surgical training (87.9% vs. 57.1%, p=0.0004).

### Compatibility with family life

At present, 67.2% (n=84) of the respondents are married or in a long-term relationship, with 33.3% (n=28) having partners who are also doctors. Thus far, 36% (n=45) of the participants have at least one child and 53.8% (n=43) plan to have children in the future (Figure 1).

Female surgeons who already have children had significantly more often completed their specialty training [66.7% (n=30) vs. 45.0% (n=36), p=0.0027] and achieved higher academic degrees [66.7% (n=30) vs. 37.5% (n=30), p=0.0253]. Both female surgeons with and without children aim at leadership positions, i.e. consultant surgeon or head of department [71.11% (n=32) vs. 66.3% (n=53), p=0.6904].

Currently, 62.3% (n=66) of the respondents live in a major city, 32.1% (n=34) in a medium-sized town, and 5.6% (n=6) in a small town. In the future, participants prefer to stay in their current place of residence [major city 61.3% (n=65), medium-sized town 30.2% (n=32), small town 8.5% (n=9); p=0.5428]. Participants living in major cities are just as likely to be in a long-term relationship [83.3% (n=55) compared to 78.4% (n=29) in more rural areas, p=0.6001]. In rural areas, female surgeons are more likely to have children [48.7% (n=19) vs. 39.4% (n=26), p=0.4161]. Female surgeons in large cities and rural areas are equally satisfied with their income [68% (n=45) vs. 75% (n=21), p=0.6247].

## Discussion

### Declining interest in surgical careers

The surgical career is unique among medical professions in combining intellectual expertise and manual skills for the treatment of patients. Nowadays, in many countries worldwide, the surgical profession is suffering from a shortage in staff as medical graduates demonstrate less interest in a surgical career. Moreover, resident attrition from surgical residency ranges from 2% to 30%; however, attrition in general surgery is not related to female gender [18]. In a 2007 survey, 17.7% of Swiss surgeons would not choose medicine as a career again [2]. The category “work-life balance” was significantly associated with not studying medicine again and “specific training conditions” was the top-ranked category against the choice of a career in surgery. Remarkably, 93.7% of the participating Swiss surgeons were men [2].

Traditionally, female medical students have been less likely to pursue a career in surgery and surgical subspecialties, as surveyed in the UK in 2009 [11]. Female residents in the US in 2016 were still found to experience a lower work-related quality of life than their male colleagues [19].

With regard to the gender shift among medical students, we decided to perform a study on female surgeons to assess their current working situation and future career strategies in order to identify opportunities guaranteeing qualified and motivated surgical trainees.

### Surgical lifestyle

Arguments against a surgical career typically concern surgical training conditions, e.g. dated curriculum, diminished caseloads, and tendency toward further subspecialties [2]. Besides, both male and female surgeons put an emphasis on work-life balance nowadays. Male surgeons are just as likely as female surgeons to report that quality of life dissuades them from choosing surgery; in fact, surgical workload and family concerns appear to be less of a deterrent for female surgeons [13], [14], [20], [21]. In a Hong Kong survey, both male and female surgeons wished for a better balance between career and home life [22]. Among American orthopedic surgeons, the most common reason proposed for why women might not choose orthopedic surgery was that orthopedic surgeons are perceived as unable to have a good work-life balance [23]. In a Canadian survey, the three worst aspects of a surgical career are reported as loss of personal time, impact on family and relationships, and time commitment [15].

### Career satisfaction

The three best aspects of being a surgeon are reported as technical skills, patient interaction, and diversity of duties [15]. Among orthopedic female surgeons, similar main reasons for choosing their career were stated: enjoyment of manual tasks, professional satisfaction, and intellectual stimulation [23]. Women who are interested in surgery are likely to pursue a surgical career despite the alleged workload and other potentially negative aspects of a surgical lifestyle [11]. Once in the surgical profession, female surgeons seem positive about their career choice and aptly fill residency positions [11]. Canadian female surgeons rated their career satisfaction as 8.6 on a scale of 1–10 [15].

In our study, the majority of the participating female surgeons aspire to become a consultant surgeon, i.e. assuming responsibility in critical clinical decision-making and guiding young professionals. Previous studies have already demonstrated that women in surgery are as eager as men to assume leadership positions, and that they are equally qualified for these positions [24]. Yet, female consultant surgeons, and even more so heads of department, remain a minority within the medical workforce. Among general surgery program directorship, men continue to hold more positions of educational leadership in the US in 2017 [25].

Female surgeons aiming at leadership positions work overtime (≥50 h/week) significantly more frequently in our study, demonstrating their willingness to compete for such openings. As more women hold academic positions in the field of general surgery, an increase in the representation in leadership is anticipated [25].

Besides having less career advancement opportunities in the field of surgery, women have been found to report less remuneration compared with their male colleagues [26], [27]. In our study, the majority (79%) rated their income as at least largely satisfying. Female surgeons satisfied with their surgical training were significantly more frequently satisfied with their income as well.

### Gender-based discrimination

Female surgeons have been known to be more concerned about gender judgment than their male colleagues [28]. Seemann et al. reported that 57% of female surgeons perceived challenges in their surgical careers as a result of their gender [15]. At least one-third of female surgeons stated that attitudes to their gender were a barrier to their career advancement [14]. As female surgeons move up through the ranks of medical student, to resident, and to staff surgeon, the perception of gender discrimination seems to increase [15]. Such a perceived dissimilar treatment could prevent female surgeons from reaching their full potential.

In a qualitative study by Webster et al., Canadian female academic surgeons shared several accounts of overt harassment and bullying as well as a subtle pressure to adopt certain rigid roles during medical training and practice [29]. However, these female surgeons claimed that gender did not enter into the organization of their workplace or into any of their professional relationships. Through disavowing and distancing themselves from gender discrimination, these women ultimately expose the degree to which these issues continue to be pervasive in surgery [29]. Despite such obstacles, many women in surgery enjoy their careers and the vast majority would make the same career choices if they went back in time [15].

In our study, those who describe statistically significantly less gender-based discrimination are more satisfied with their surgical training. Although female surgeons are generally satisfied with their careers, more American women exit academic surgery at early career stages due to gender discrimination, lack of opportunities for career advancement, and networking difficulties resulting from a paucity of senior female mentors [14]. Hence, measures to address gender-based discrimination in the surgical workplace remain important.

### Impact on family life

In a survey among senior surgical residents and early career surgery faculty members in the United States, female surgeons were less likely to marry and more likely to significantly delay or altogether forgo childbearing; 43% of female surgeons agreed that having children would be a career barrier [14]. In Canada, 89% of female surgeons in academic centers were in a long-term relationship and 79% had at least one child. Nevertheless, participants felt that balancing their surgical careers with family commitments was a major source of stress [15]. Among Austrian women in leadership positions, it has been shown that inadequate help with child care has been a barrier to career advancement [30]. In addition, there still seem to be societal expectations that make it challenging for women to balance a successful career and family life. In our study, 36% of female surgeons have children and 54% wish to have children in the future. Most (67%) of the respondents with children had completed their specialty training and obtained higher academic degrees. Apparently, female surgeons aim at achieving professional development before realizing parenthood.

### Inspiration for the future

Satisfaction with career advancement is significantly higher in women with a mentor-mentee relationship, as found in a Swiss study [16]. Mentorship seems to be more frequently available in North America compared to Europe. In a Canadian survey, 79% of participants identified at least one mentor, 89% of these mentors being men [15]. Many participants wished they had women as mentors; the benefit of having a female mentor would be for advice on how to balance career with family life [15]. Likewise, American female medical students emphasized the importance of female mentorship and organizations supporting women in surgery to apply for a surgical residency [31]. When selecting a general surgery residency program, American women top-rank the same criteria as men, i.e. variety/number of cases, friendly training environment, and quality of relationships with attending physicians. The selection criteria receiving significantly higher scores among women are the number of female residents and number of female consultants [32]. The presence of female surgical role models is essential in encouraging female medical students to not only enter the field of surgery but also to take on higher academic roles in this field [31].

### Limitations

The main strength of our study is that it was conducted among surgeons in different career settings and work places. Due to the methodological setting of a questionnaire-based study, the results are subject to responder bias. Our response rate of 40.8% has to be highlighted for this type of study, as it is well known that physicians are often a group with low survey response rates. An overall survey response rate among physician specialists of 35% and in general surgery of 29% is common [33]. The current study focused on women self-reporting surgical career strategies; therefore, male surgeons were excluded. Gender differences in career choices and advancement may be identified through further research including male surgeons. In the literature, there is a paucity of data comparing career aspirations of male and female surgeons as well. Twenty years ago, Schroen et al. evaluated the professional experiences of male (53.0%) and female (47.0%) members of the American College of Surgeons [27]. At that time, men had expressed higher ultimate goals for academic rank than women; e.g. 25% of men declared chairmanship to be their ultimate goal compared with 5% of women. In a previous survey of our group focusing on workload and professional satisfaction of male (66.1%) and female (33.9%) transplant specialists of different medical disciplines, more than two-thirds had already reached potential career goals, i.e. leadership positions [34]. Thus, an assessment of career aspirations was not performed.

## Conclusions

Female surgeons seem positive about their career choice and will aptly fill any upcoming leadership positions. Women interested in surgery are likely to pursue a surgical career, demonstrating the importance of professional development among female surgeons. To attract medical graduates regardless of gender to surgical careers and to retain talented surgeons in their profession, a structured curriculum, recognition at work, strategies to improve work-life balance, and family planning remain important.
