# Supplementary material for: Women in surgery: a web-based survey on career strategies and career satisfaction
Source: Innov Surg Sci. 2020 Feb 29;5(1-2):11–9. doi: 10.1515/iss-2019-0016 (PMC7798307; doi:10.1515/iss-2019-0016)
Supplement: Supplementary file 1 [file iss-5-20190016-s001.pdf]

Reviewer Assessment

Sonia Radunz*, Hülya Pustu, Katja Marx, Laura Mazilescu, Agnes Braun, Tamas Benkö, Mark Banysch and Gernot M. Kaiser

Women in surgery: a web-based survey on career strategies and career satisfaction

https://doi.org/10.1515/iss-2019-0016
Received November 18, 2019; accepted January 23, 2020

***Corresponding author: PD Dr. med. Sonia Radunz,** Department of General, Visceral and Transplant Surgery, University Hospital Essen, Hufelandstr. 55, 45147 Essen, Germany, Phone: +49-201-723-84019, Fax: +49-201-723-1113; and Department of General, Visceral and Transplant Surgery, University Hospital Münster, Alber-Schweitzer-Campus 1, 48140 Münster, Germany, Phone: +49-251-8351765, E-mail: sonia.radunz@uk-essen.de. https://orcid.org/0000-0002-3874-1336
Hülya Pustu, Katja Marx, Mark Banysch and Gernot M. Kaiser: Department of General and Visceral Surgery, St. Bernhard-Hospital Kamp-Lintfort, Kamp-Lintfort, Germany
Laura Mazilescu and Tamas Benkö: Department of General, Visceral and Transplant Surgery, University Hospital Essen, Essen, Germany
Agnes Braun: MVZ Media Vita, Doctor's Office for Surgery, Issum, Germany

Reviewers' Comments to Original Submission

Reviewer 1: Frauke Fritze-Büttner

Dec 03, 2019

**Reviewer Recommendation Term:**	**Accept**
**Overall Reviewer Manuscript Rating:**	**40**
Is the subject area appropriate for you?	5 - High/Yes
Does the title clearly reflect the paper's content?	5 - High/Yes
Does the abstract clearly reflect the paper's content?	5 - High/Yes
Do the keywords clearly reflect the paper's content?	4
Does the introduction present the problem clearly?	5 - High/Yes
Are the results/conclusions justified?	5 - High/Yes
How comprehensive and up-to-date is the subject matter presented?	5 - High/Yes
How adequate is the data presentation?	4
Are units and terminology used correctly?	4
Is the number of cases adequate?	4
Are the experimental methods/clinical studies adequate?	5 - High/Yes
Is the length appropriate in relation to the content?	4
Does the reader get new insights from the article?	4
Please rate the practical significance.	5 - High/Yes
Please rate the accuracy of methods.	5 - High/Yes
Please rate the statistical evaluation and quality control.	5 - High/Yes
Please rate the appropriateness of the figures and tables.	3
Please rate the appropriateness of the references.	4
Please evaluate the writing style and use of language.	4
Please judge the overall scientific quality of the manuscript.	4
Are you willing to review the revision of this manuscript?	Yes

Open Access. © 2020 Radunz S., et al., published by De Gruyter. This work is licensed under the Creative Commons Attribution 4.0 Public License.

Comments:
This work sheds light on an important occupational policy issue. Despite the increasing feminization of surgical disciplines, women are largely under-represented in management positions. On the one hand, women no longer necessarily aspire to a managerial function after starting a family and fulfilling their wishes, on the other hand, the way to fulfill this is more demanding for them and usually only possible with a significant additional effort. The study shows the results of a survey in North Rhine-Westphalia. The response rate is very good at over 40%, but the total number of participants is low. Irrespective of this, it is safe to say that structured education and training, personal recognition and appreciation in everyday work and an improved work- life- family- balance are essential prerequisites for medical graduates, regardless of gender, to pursue a career in surgery.

Reviewer 2: anonymous

Dec 27, 2019

**Reviewer Recommendation Term:**	**Accept**
**Overall Reviewer Manuscript Rating:**	**65**
Is the subject area appropriate for you?	5 - High/Yes
Does the title clearly reflect the paper's content?	3
Does the abstract clearly reflect the paper's content?	4
Do the keywords clearly reflect the paper's content?	4
Does the introduction present the problem clearly?	4
Are the results/conclusions justified?	4
How comprehensive and up-to-date is the subject matter presented?	5 - High/Yes
How adequate is the data presentation?	3
Are units and terminology used correctly?	- N/A
Is the number of cases adequate?	3
Are the experimental methods/clinical studies adequate?	4
Is the length appropriate in relation to the content?	4
Does the reader get new insights from the article?	3
Please rate the practical significance.	4
Please rate the accuracy of methods.	- N/A
Please rate the statistical evaluation and quality control.	4
Please rate the appropriateness of the figures and tables.	4
Please rate the appropriateness of the references.	5 - High/Yes
Please evaluate the writing style and use of language.	4
Please judge the overall scientific quality of the manuscript.	3
Are you willing to review the revision of this manuscript?	Yes

Comments to Author:
None
